# Serum Creatinine Level and APACHE-II Score within 24 h of Admission Are Effective for Predicting Persistent Organ Failure in Acute Pancreatitis

**DOI:** 10.1155/2019/8201096

**Published:** 2019-03-10

**Authors:** Jianhua Wan, Wenqing Shu, Wenhua He, Yin Zhu, Yong Zhu, Hao Zeng, Pi Liu, Liang Xia, Nonghua Lu

**Affiliations:** Department of Gastroenterology, The First Affiliated Hospital of Nanchang University, Nanchang, China

## Abstract

**Aim:**

The present study was aimed at comparing serum markers and APACHE-II score to predict persistent organ failure (POF) in early acute pancreatitis (AP).

**Methods:**

In this retrospective study, data from 6024 patients with AP were included within 24 h of their admission. Serum levels of urea nitrogen (BUN), creatinine, glucose, and hematocrit and APACHE-II score were analyzed for patients with AP. We employed the area under the receiver operating characteristic (ROC) curve (AUC), sensitivity, and specificity analyses to evaluate the accuracy of the studied laboratory parameters and APACHE-II score.

**Results:**

Our study included 2090 (35%) patients out of 6024 patients who were evaluated within 24 h of hospital admission. For predicting POF, serum creatinine level ≥ 1.8 mg/dl had the highest specificity (98%). The second classification tree has shown that when the serum creatinine level > 1.8 and APACHE − II ≥ 8 within 24 h were combined, the rates of predicted persistent organ failure achieved 66.7%.

**Conclusions:**

In this large, hospital-based retrospective study, we demonstrated that an APACHE-II score ≥ 8 and a serum creatinine level ≥ 1.8 mg/dl within 24 h of admission can positively predict POF in AP and that serum creatinine levels < 1.8 mg/dl within 24 h of admission can be useful for negatively predicting POF in AP.

## 1. Introduction

Over the last several decades, acute pancreatitis (AP) has emerged as one of the most common reasons for hospitalization in individuals with gastrointestinal conditions [1].^.^Most of the AP patients present with mild acute pancreatitis (MAP) and have good prognosis; however, 15-20% of AP patients develop severe acute pancreatitis (SAP), which results in significant morbidity and mortality [2].

The most lethal complication of AP is persistent organ failure (POF) in early phases of the disease. Thus, it is very important to effectively predict POF during the initial stages of AP and to determine whether patients may need to be timely transferred to the intensive care unit (ICU) for treatments including adequate initial fluid resuscitation. Many previous studies have reported that a series of available scoring systems, including the Acute Physiology, Chronic Health Evaluation II (APACHE-II) score, Ranson score, modified Glasgow/Imrie score, systemic inflammatory response syndrome (SIRS) criteria, Bedside Index for the Severity in Acute Pancreatitis (BISAP) score, and Harmless Acute Pancreatitis Score (HAPS), can be used for the prediction of POF in clinical practice. Notably, the main limitation of these scoring systems is that these are difficult to widely practice during clinical management. Furthermore, Mounzer et al. have found that all of these clinical scoring systems are moderately accurate in predicting POF and that the accuracy of various predictive scoring systems is comparable [3]. Therefore, none of the scoring system can be used to produce satisfactory results.

Given that the accuracy of various predictive scoring systems is similar, current research is exploring simple, accurate, inexpensive, and repeatable methods to predict the severity of AP. Over the last two decades, a number of studies have reported that the serum levels of biochemical markers (blood urea nitrogen (BUN), creatinine, glucose, and hematocrit) within 24-48 h of admission are closely associated with the severity of AP [4–10]. Most investigators claim that they have identified accurate laboratory parameters to predict the severity of AP. However, most of these studies lack adequate sample size and data from the comparison of different biochemical markers in the same group of patients. In addition, based on previous evidence, the APACHE-II score is the most accurate predictive index [3, 11]. Therefore, we performed this retrospective study on a large sample in a single center.

The aims of the present study were (1) to compare the early serum levels of BUN, creatinine, glucose, and hematocrit and APACHE-II score for the prediction of POF in AP and (2) to determine whether the combination of a highly precise scoring system with a simple laboratory parameter can significantly improve the accuracy of predicting POF in AP.

## 2. Methods

### 2.1. Study Design and Patient Population

From January 1, 2005, and December 31, 2016, we performed a retrospective single-center observational study on 2090 patients within 24 h of their admission to our hospital. The study was approved by the ethics committee of The First Affiliated Hospital of Nanchang University (No. 2011001).

### 2.2. Patient Selection

All data were obtained from an electronic medical database. The inclusion criteria were as follows: (1) patients diagnosed with AP at our hospital within 24 h of admission; (2) evaluation of serum levels of BUN, creatinine, glucose, and hematocrit and the APACHE-II score within 24 h of admission; and (3) available data on patient age and sex, AP etiology and severity, presence of complications, length of hospital stay, and mortality.

### 2.3. Definitions

AP was diagnosed according to the 2012 version of the Atlanta Classification System and internationally accepted definitions based on the presence of at least two of the following three criteria [12]: (1) classic abdominal pain; (2) elevation of serum amylase and/or lipase level to three times the upper limit of the normal range; and (3) radiographic evidence of AP. The classification of SAP and moderately severe AP (MSAP) were also based on the 2012 version of the Atlanta Classification System and internationally accepted definitions [12]. Organ failure comprised the cardiovascular, pulmonary, and/or renal systems and was defined as persistent systolic blood pressure < 90 mm Hg following fluid resuscitation, arterial PO_2_ < 60 mm Hg in room air or the requirement for mechanical ventilation, and/or a serum creatinine level ≥ 2 mg/dl after rehydration or the need for hemodialysis in patients without preexisting renal disease, respectively. Organ failure was denoted POF when it lasted longer than 48 h.

### 2.4. Statistical Analysis

Statistical analyses were performed using IBM SPSS software, version 20.0 (Statistical Package for the Social Sciences, Chicago, IL, USA). Sensitivity, specificity, positive predictive value, and negative predictive value were calculated for the serum levels of BUN, creatinine, glucose, and hematocrit and the APACHE-II score at admission. Predictive accuracy was measured by the area under the receiver operating characteristic (ROC) curve (AUC).

## 3. Result

### 3.1. Patient Demographics and Clinical Characteristics

In total, 2090 (35%) patients out of 6024 patients were evaluated within 24 h of hospital admission. Demographic and clinical characteristics (age, sex, AP etiology, comorbidities, and mortality) were similar between patients grouped based on the time after admission (<24 h vs. >24 h) as shown in Table 1. Hypertriglyceridemia was more common as an etiology of AP in patients evaluated <24 h after admission than in patients evaluated >24 h after admission (25% vs. 17%; *P* < 0.001), and this phenomenon seemed be associated with metabolic characteristics including elevated triglyceride levels and history of hypertriglyceridemia (9% vs. 5%; *P* < 0.001). Slight differences in the presence of pancreatic necrosis (17% vs. 21%; *P* < 0.001), proportion of cases with SAP (15% vs. 17%; *P* = 0.083), and mortality (1% vs. 1.3%; *P* < 0.001) between the two groups may be related to timely and effective treatment associated with early admission. No significant difference was observed between the groups regarding age, etiology related to alcohol use, and the proportion of idiopathic AP.

The median age of the 2090 patients evaluated within 24 h of hospital admission was 51 years (interquartile range 40-63 years), and 58% of the patients were male. Classification of patients according to AP etiology revealed that 1173 (56%) AP cases were of biliary correlation, 130 (6%) were associated with alcohol abuse, 531 (25) were associated with hypertriglyceridemia, and 185 (9%) cases were idiopathic.

### 3.2. Comparison of Performance among Serum Levels of BUN, Creatinine, Glucose, and Hematocrit and APACHE-II Score at Admission in Predicting POF

Table 2 summarizes the performance of BUN level ≥ 20 mg/dl, creatinine level ≥ 1.8 mg/dl, glucose level ≥ 7.1 mmol/l, hematocrit level ≥ 44%, and APACHE-II score ≥ 8 within 24 h of admission for the prediction of POF. For predicting POF, APACHE-II scores ≥ 8 had the highest accuracy with an AUC of 0.67, serum creatinine level ≥ 1.8 mg/dl had the highest specificity (98%) and positive predictive value (60%) with an AUC of 0.58, and glucose level ≥ 7.1 mmol/l had the highest sensitivity (74%) and negative predictive value (92%) with an AUC of 0.63.

### 3.3. Comparison of Performance among Serum Levels of BUN, Creatinine, Glucose, and Hematocrit and APACHE-II Score at Admission for the Prediction of POF in AP of Different Etiologies

As shown in Tables 3–7, serum BUN level ≥ 20 mg/dl and creatinine level ≥ 1.8 mg/dl had the highest accuracy in predicting POF in alcohol-induced AP, with AUCs of 0.75 and 0.68, specificities of 58% and 37%, sensitivities of 92% and 99%, positive predictive values of 58% and 88%, and negative predictive values of 92% and 88%, respectively. Regarding idiopathic AP, glucose level ≥ 7.1 mmol/l and APACHE-II score ≥ 8 had the highest accuracy in predicting POF, with AUCs of 0.69 and 0.65, specificities of 80% and 47%, and sensitivities of 57% and 84%, respectively. APACHE-II score ≥ 8 was the most accurate in predicting POF in AP of biliary origin and hypertriglyceridemia-associated AP, with AUCs of 0.67 and 0.67, specificities of 60% and 49%, and sensitivities of 75% and 84%, respectively. Finally, serum creatinine level < 1.8 mg/dl assessed within 24 h of admission had the highest specificity in predicting POF in AP of different etiologies (98%, 99%, 97%, and 95%), as shown in Table 4.

### 3.4. Outperformance of APACHE-II score ≥ 8 plus Serum Creatinine Level ≥ 1.8 mg/dl over Serum Hematocrit Level > 44% plus Serum BUN Level ≥ 20 mg/dl at Admission Based on an Early Prediction Classification Tree

**Figure 1:**
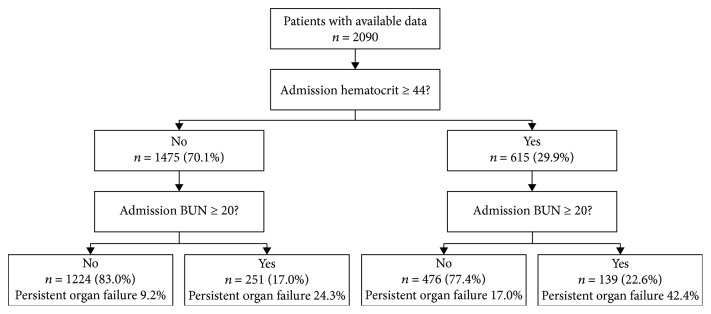
Classification tree predicting persistent organ failure and pancreatic necrosis based on hematocrit (HCT) level ≥ 44% on admission and rise in serum urea nitrogen (BUN) level ≥ 20% at 24 h.

**Figure 2:**
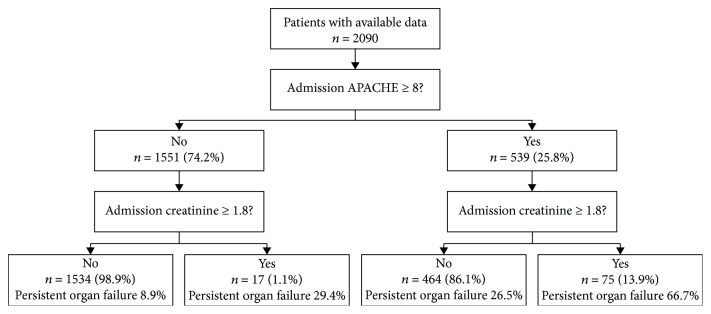
Classification tree predicting persistent organ failure and pancreatic necrosis based on APACHE − II ≥ 8 on admission and serum creatinine level ≥ 1.8 at 24 h.

Koutroumpakis et al. constructed an early prediction classification tree based on hematocrit level > 44% and increased BUN levels at admission [13]. However, in our cohort, POF developed in 42.4% of patients with hematocrit level ≥ 44% plus BUN level ≥ 20 mg/dl within 24 h of admission and in 9.2% of patients with hematocrit level < 44% and BUN level < 20 mg/dl within 24 h of admission (Figure 1). Thus, we investigated whether APACHE-II score ≥ 8 and serum creatinine level ≥ 1.8 mg/dl may be better at predicting POF in AP than were the other indices. Of the 2090 patients with AP, 539 had APACHE-II scores ≥ 8 at admission. Of those 539 patients, 73 had serum creatinine levels ≥ 1.8 mg/dl within 24 h of admission, and among them, 50 (66.7%) developed POF. Overall, 464 patients had elevated APACHE-II scores at admission without elevated serum creatinine levels; among these patients, 123 (26.5%) developed POF. A total of 1551 patients had APACHE-II scores < 8 at admission; among these patients, 17 had serum creatinine levels ≥ 1.8 mg/dl within 24 h of admission, and among them, 5 (29.4%) developed POF. In total, 1534 patients had both APACHE-II scores < 8 and serum creatinine levels < 1.8 mg/dl at admission. The incidence of POF among these patients was 8.9% (*n* = 137; Figure 2).

## 4. Discussion

We performed a retrospective single-center study on a large sample to assess the accuracy of POF prediction based on early laboratory parameters. Our data indicated that single laboratory indices do not have high accuracy for predicting POF in AP. The data in Table 2 demonstrate that the accuracy of all biochemical parameters is limited by the corresponding sensitivities and specificities. Although glucose level ≥ 7.1 mmol/l was the most accurate (AUC = 0.63) among all laboratory indices in predicting POF, it was still inferior to the APACHE-II score ≥ 8 (AUC = 0.67). Thus, the combination of clinical scoring systems with laboratory indices may be effective for improving overall predictive accuracy.

Previous studies have reported that early changes in serum creatinine levels in AP are useful indicators of disease severity and mortality [6, 8]. One study demonstrated serum creatinine level within 48 h of admission as a highly accurate predictor of pancreatic necrosis in AP (AUC = 0.77) [6]. Another retrospective study demonstrated that serum creatinine level within 48 h of admission is a good predictor of fatal AP (AUC = 0.879) [8]. However, Lankisch et al. reported that an elevated serum creatinine level during the first 48 h of admission is not a marker for pancreatic necrosis in the initial stages of AP [14]. However, that study had a flaw in which the diagnosis of pancreatic necrosis was made based on contrast-enhanced computed tomography (CT) performed within 96 h instead of after 7 days, because it may underestimate the eventual extent of pancreatic and peripancreatic necrosis [2]. Furthermore, their research did not study the relationship between serum creatinine and infected necrosis, because it is usually rare during the first week [15]. These findings indicate that serum creatinine level might be a good laboratory index for predicting POF in the early phases of AP.

The relationship between the severity of AP and BUN levels has been extensively studied in recent years. Wu et al. have demonstrated BUN level as the most accurate predictor of mortality among indicators including calcium, hemoglobin, creatinine, and glucose levels [4]. Another study demonstrated significant associations between elevated BUN levels at admission and prolonged ICU stay and mortality in patients [16]. BUN is one of the common indicators of renal function, and BUN levels increase during renal failure. Therefore, in theory, BUN levels may be a predictor of persistent renal failure.

Furthermore, many studies have reported close associations of elevated hematocrit levels within 24 h of admission with organ failure and necrotizing pancreatitis [9, 17]. Based on our results, hematocrit concentration had a sensitivity and specificity of 87% and 65%, respectively, for predicting POF [9]. Moreover, a retrospective analysis found that a 5% increase in hematocrit concentration was a significant predictor of severe pancreatitis (odds ratio (OR) = 2.8; *P* = 0.001) and necrosis (OR = 3.9; *P* = 0.001) [17]. However, Lankisch et al. demonstrated that hematocrit concentration was not significantly correlated with organ failure or mortality rate [18], although the sample size in that trial was not large, and the cutoff for hematocrit level was relatively low (43% for males and 39.6% for females). Thus, hematocrit level has the potential to be used as a positive predictor of POF.

The association between glucose level and the severity of AP has also been previously reported. A study evaluated 170 patients with AP, and ROC analysis showed significant associations of glucose levels at admission with local complications (*P* = 0.009), systemic complications (*P* = 00001), and mortality (*P* = 0.0001) [10]. In another study, using logistic regression analysis, Mentula et al. proposed serum glucose level as an independent predictor of organ failure [19]. However, one prospective study found that high blood glucose levels were significantly correlated with total hospital stay but not with organ failure or mortality [20]. In that study, organ failure was measured using prognostic scores rather than the modified Marshall scoring system, and the level of cutoff for glucose was lower than that in the previous article. Thus, blood glucose level may be used as a positive predictor of POF.

Other scoring systems may also be helpful in predicting organ failure. Based on current evidence, the APACHE-II score has the following advantages in predicting organ failure. First, APACHE-II scores ≥ 8 are more accurate in predicting organ failure than are other scoring systems [3, 11]. Second, the APACHE-II score is widely used by clinicians. Finally, the APACHE-II score can be used to assess changes in each physiological parameter to reflect the condition of the body in a more realistic and comprehensive manner. Nevertheless, despite these advantages, the APACHE-II score alone should not be used by physicians for predicting POF.

The above studies have highlighted promising strategies for predicting the severity of AP. However, our study revealed that laboratory parameters are only moderately accurate in predicting POF with modest AUCs and that their efficacy is not as high as reported in the previous articles [4–10]. Nevertheless, we believe that our study has provided more reliable evidence than previous studies regarding POF prediction. First, we used a large sample size and compared the same biochemical markers in patients with AP of different etiologies, both of which were lacking in previous studies. In addition, the severity of AP in previous studies was assessed using various methods rather than the more rigorous and accurate Atlanta Classification System that has been used in our study. Thus, those studies may have had different degrees of selection bias. We avoided this problem by using the latest revised definitions and classification of AP. Additionally, although combining all existing clinical scores and laboratory test results may significantly improve the predictive accuracy for POF, this method may not be practical for clinical application, as it is complex and inconvenient. Thus, the combination of a highly precise scoring system and a simple laboratory parameter may be a good solution for this problem. In the present study, serum creatinine level and APACHE-II score at admission were the most accurate predictors of POF. Moreover, based on our classification tree, when both APACHE − II score ≥ 8 and serum creatinine level ≥ 1.8 mg/dl within 24 h of admission were combined, 66.7% of patients with AP were predicted to progress to POF, which is higher than the proportions shown in previous studies [13].

Based on above results, until a better approach has been identified, serum creatinine levels and APACHE-II scores obtained within 24 h of admission can help clinicians to predict the occurrence of POF and to decide on early management strategies. Early and vigorous fluid resuscitation maintains microcirculation, which may improve the prognosis of patients with SAP. Some recently published studies have proposed that early vigorous fluid resuscitation might prevent pancreatic necrosis and reduce the incidence of SIRS and organ failure at the 72 h timepoint, and it might even decrease in-hospital mortality [21–23].

In addition, an important finding of our study is that there is a very low likelihood of developing POF in AP patients who have a serum creatinine level < 1.8 mg/dl within 24 h of admission. The hypovolemia caused by various causes in AP will first lead to a deficiency of renal perfusion, and it will further develop into renal failure. If a patient has a serum creatinine level < 1.8 mg/dl within 24 h of admission, it indicates that their renal function is acceptable, as are their other organ functions. In general, these patients do not require treatment in the ICU or vigorous fluid resuscitation. Moreover, some studies have suggested that aggressive hydration can increase the likelihood of complications and mortality [24, 25].

This study has several limitations. First, all the data were retrospectively analyzed. Second, we included patients within 24 h of disease onset and excluded patients with more than 24 h of onset, which may have led to selection bias. Third, serum creatinine levels and APACHE-II scores were not recorded in our hospital database for all patients within 24 h of admission, and thus, unexpected biases may have arisen. To increase the reliability of our results and to reduce these potential biases, we need even larger samples. The present study provides preliminary data for the design of future randomized controlled trials for predicting POF during the initial stages of AP.

In conclusion, the combination of APACHE-II score ≥ 8 and serum creatinine level ≥ 1.8 mg/dl within 24 h of admission is the most accurate at predicting POF. Additionally, serum creatinine level < 1.8 mg/dl within 24 h of admission had excellent specificity (98%) in excluding the possibility of POF. Thus, patients with serum creatinine level < 1.8 mg/dl within 24 h of admission likely do not need to be treated in the ICU. Hence, our findings may be useful for physicians for the initial treatment of patients with AP.

## Figures and Tables

**Table 1:** Population baseline characteristics between AP patients within 24 h of admission vs. those over 24 h.

Variable	AP within 24 h	AP over 24 h	*P*
	*N* = 2090	*N* = 3934	
Median age (years) (IQR)	51 (40-63)	51 (40-63)	0.957
Sex, *N* (%)			<0.001
Male	1207 (58)	2025 (52)	
Female	883 (42)	1909 (48)	
Pancreatitis etiology, *N* (%)			
Biliary	1173 (56)	2466 (63)	<0.001
Alcoholism	130 (6)	248 (6)	0.911
Hypertriglyceridemia	531 (25)	656 (17)	<0.001
Idiopathic	185 (9)	398 (10)	0.114
Comorbidities, *N* (%)			
Posthypertriglyceridemia	195 (9)	196 (5)	<0.001
Diabetes mellitus	166 (8)	253 (6)	0.028
Pancreatic necrosis	352 (17)	834 (21)	<0.001
Severity classification, *N* (%)			
MAP	970 (46)	1701 (43)	0.017
MSAP	806 (39)	1574 (40)	0.275
SAP	314 (15)	659 (17)	0.083
Hospital LOS (days) (IQR)	8 (6-12)	9 (6-13)	0.001
Mortality (%)	1.0	1.4	<0.001

AP, acute pancreatitis; *N*, number; IQR, interquartile range.

**Table 2:** Admission serum BUN, creatinine, glucose, and hematocrit levels and APACHE-II score as markers for persistent organ failure.

Laboratory markers	Persistent organ failure		Sensitivity (%)	Specificity (%)	Positive predictive value (%)	Negative predictive value (%)	AUC
	Yes	No					
BUN							
≥20	120	270	38	85	31	89	62
<20	194	1506					
Creatinine							
≥1.8	55	37	18	98	60	87	58
<1.8	259	1739					
Glucose							
≥7.1	233	845	74	52	22	92	63
<7.1	81	931					
Hematocrit							
≥44	140	475	45	73	23	88	59
<44	174	1301					
APACHE-II							
≥8	173	366	55	79	32	91	67
<8	141	1410					

**Table 3:** Admission serum BUN level vs. the outcome of persistent organ failure by etiology of acute pancreatitis.

BUN	Persistent organ failure		Sensitivity (%)	Specificity (%)	Positive predictive value (%)	Negative predictive value (%)	AUC
	Yes	No					
Biliary							
≥20	66	172	39	83	28	89	61
<20	102	832					
Alcoholic							
≥20	11	8	58	92	58	92	75
<20	8	96					
Hypertriglyceridemia							
≥20	23	31	30	88	43	81	59
<20	54	232					
Idiopathic							
≥20	3	27	20	83	10	92	52
<20	12	134					

**Table 4:** Admission serum creatinine level vs. the outcome of persistent organ failure by etiology of acute pancreatitis.

Creatinine	Persistent organ failure		Sensitivity (%)	Specificity (%)	Positive predictive value (%)	Negative predictive value (%)	AUC
	Yes	No					
Biliary							
≥1.8	23	15	14	98	61	87	56
<1.8	145	989					
Alcoholic							
≥1.8	7	1	37	99	88	90	68
<1.8	12	103					
Hypertriglyceridemia							
≥1.8	16	8	21	97	67	81	59
<1.8	61	255					
Idiopathic							
≥1.8	1	8	7	95	11	92	51
<1.8	14	153					

**Table 5:** Admission serum glucose level vs. the outcome of persistent organ failure by etiology of acute pancreatitis.

Glucose	Persistent organ failure		Sensitivity (%)	Specificity (%)	Positive predictive value (%)	Negative predictive value (%)	AUC
	Yes	No					
Biliary							
≥7.1	118	441	70	56	21	92	63
<7.1	50	563					
Alcoholic							
≥7.1	15	50	79	52	23	93	65
<7.1	4	54					
Hypertriglyceridemia							
≥7.1	67	181	87	31	27	89	59
<7.1	10	82					
Idiopathic							
≥7.1	12	69	80	57	15	97	69
<7.1	3	92					

**Table 6:** Admission hematocrit level vs. the outcome of persistent organ failure by etiology of acute pancreatitis.

HCT	Persistent organ failure		Sensitivity (%)	Specificity (%)	Positive predictive value (%)	Negative predictive value (%)	AUC
	Yes	No					
Biliary							
≥44	71	197	42	80	26	89	61
<44	97	807					
Alcoholic							
≥44	12	47	63	54	20	89	59
<44	7	57					
Hypertriglyceridemia							
≥44	38	124	49	47	23	78	51
<44	39	139					
Idiopathic							
≥44	6	49	40	70	11	93	55
<44	9	112					

**Table 7:** Admission APACHE-II score vs. the outcome of persistent organ failure by etiology of acute pancreatitis.

APACHE-II	Persistent organ failure		Sensitivity (%)	Specificity (%)	Positive predictive value (%)	Negative predictive value (%)	AUC
	Yes	No					
Biliary							
≥8	100	254	60	75	28	92	67
<8	68	750					
Alcoholic							
≥8	10	13	53	87	43	91	70
<8	9	91					
Hypertriglyceridemia							
≥8	38	43	49	84	47	85	67
<8	39	220					
Idiopathic							
≥8	7	26	47	84	21	94	65
<8	8	135					

## Data Availability

All data generated or analyzed during this study are included in this published article.

## Abbreviations

BUN: Blood urea nitrogen
POF: Persistent organ failure
AP: Acute pancreatitis
ROC: Receiver operating characteristic
MAP: Mild acute pancreatitis
SAP: Severe acute pancreatitis
ICU: Intensive care unit
APACHE-II: Acute physiology, chronic health evaluation II
SIRS: Systemic inflammatory response syndrome
BISAP: Bedside index for the severity in acute pancreatitis
HAPS: Harmless acute pancreatitis score
MSAP: Moderately severe AP
CT: Computed tomography.
